# Objectively Measured Activity Patterns among Adults in Residential Aged Care

**DOI:** 10.3390/ijerph10126783

**Published:** 2013-12-04

**Authors:** Natasha Reid, Elizabeth Eakin, Timothy Henwood, Justin W. L. Keogh, Hugh E. Senior, Paul A. Gardiner, Elisabeth Winkler, Genevieve N. Healy

**Affiliations:** 1 Cancer Prevention Research Centre, School of Population Health, The University of Queensland, Brisbane, QLD 4072, Australia; E-Mails: e.eakin@sph.uq.edu.au (E.E.); e.winkler@sph.uq.edu.au (E.W.); g.healy@sph.uq.edu.au (G.N.H.); 2 Blue Care Research & Practice Development Centre, School of Nursing and Midwifery, University of Queensland, Brisbane, QLD 4072, Australia; E-Mail: t.henwood@uq.edu.au; 3 Faculty of Health Sciences and Medicine, Bond University, Robina, QLD 4226, Australia; E-Mail: jkeogh@bond.edu.au; 4 Human Potential Centre, Auckland University of Technology, Auckland 1010, New Zealand; 5 Cluster for Health Improvement, Faculty of Science, Health, Education and Engineering, University of the Sunshine Coast, Sippy Downs, QLD 4556, Australia; 6 Discipline of General Practice, School of Medicine, University of Queensland, Brisbane, QLD 4072, Australia; E-Mail: h.senior@uq.edu.au; 7 Centre for Longitudinal and Life Course Research, School of Population Health, The University of Queensland, Brisbane, QLD 4072, Australia; E-Mail: p.gardiner@sph.uq.edu.au; 8 Translating Research into Practice (TRIP) Centre, Mater Research, South Brisbane, QLD 4101, Australia; 9 Baker IDI Heart & Diabetes Institute, Melbourne, VIC 3004, Australia; 10 School of Physiotherapy, Curtin University, Bentley, WA 6102, Australia

**Keywords:** older adults, physical activity, sedentary time, sitting-objective measurement, activPAL

## Abstract

*Objectives:* To determine the feasibility of using the activPAL3^TM^ activity monitor, and, to describe the activity patterns of residential aged care residents. *Design:* Cross-sectional. *Setting:* Randomly selected aged care facilities within 100 km of the Gold Coast, Queensland, Australia. *Participants:* Ambulatory, older (≥60 years) residential aged care adults without cognitive impairment. *Measurements:* Feasibility was assessed by consent rate, sleep/wear diary completion, and through interviews with staff/participants. Activity patterns (sitting/lying, standing, and stepping) were measured via activPAL3^TM^ monitors worn continuously for seven days. Times spent in each activity were described and then compared across days of the week and hours of the day using linear mixed models. *Results:* Consent rate was 48% (n = 41). Activity patterns are described for the 31 participants (mean age 84.2 years) who provided at least one day of valid monitor data. In total, 14 (45%) completed the sleep/wear diary. Participants spent a median (interquartile range) of 12.4 (1.7) h sitting/lying (with 73% of this accumulated in unbroken bouts of ≥30 min), 1.9 (1.3) h standing, and 21.4 (36.7) min stepping during their monitored waking hours per day. Activity did not vary significantly by day of the week (*p* ≥ 0.05); stepping showed significant hourly variation (*p* = 0.018). *Conclusions:* Older adults in residential aged care were consistently highly sedentary. Feasibility considerations for objective activity monitoring identified for this population include poor diary completion and lost monitors.

## 1. Introduction

Over recent years there has been a rapid uptake and use of device-based measures to capture and estimate physical activity and sedentary time [1]. Use of such monitors within large, population-based studies have highlighted that older adults are one of the least physically active [2] and the most sedentary [3,4] population groups. This poor activity profile places them at increased risk for the negative health consequences associated with inactivity and prolonged sedentary time [5,6,7]. However, studies typically recruit community-dwelling older adults [3,8], with those living in residential aged care (RAC) being understudied. This rapidly escalating population group [9] (e.g., 25% increase in permanent aged care residents over the last decade in Australia [10]), incur substantial economic burden [10] and are an important target group for interventions to improve functional capacity and well-being. Although activity levels are a key factor for sustained health and reduced premature mortality [11], to date, there has been minimal investigation into the activity patterns of adults living in RAC. Further, the feasibility of using device-based activity monitoring is a concern for this population group as they are likely to suffer cognitive decline [12], depression [13], and possible skin irritation [14,15], resulting in difficulty complying with protocols, and/or refusal to participate. 

Therefore, the aims of this study were: (1) to evaluate the feasibility of using an activity monitor in a RAC sample; and, (2) to estimate the sitting, standing and stepping patterns of older adults in RAC. Activity patterns were examined as a weekly average, as well as across days of the week and hours of the day, and in terms of how sitting time in particular, is accrued. This information is important, not only for understanding the scope of the problem (How sedentary and/or inactive are adults in RAC?), but also for informing future interventions (Is it feasible/when is the best time to intervene?), and monitoring protocols (How many days of monitoring are required?).

## 2. Methods

### 2.1. Context

This research (Activity Patterns Study) was conducted as a sub-study of a larger investigation of the prevalence and risk factors of sarcopenia in a randomly recruited sample of older adults in RAC. All aged care centres that were part of a specific RAC facility organisation with a large number of residents of varying care levels and that were within a 100 km radius of the Gold Coast, Australia, were selected for the larger study. All eleven RAC centres within the inclusion zone agreed to participate. Residents were excluded if they were younger than 60 years, had a pacemaker, or were end-stage palliative or terminal. Behavioural problems that would endanger the research assistant or resident during data collection or a medical or other issue that would make them difficult to work with, including total uncommunicable deafness and diagnosed severe dementia, were also grounds for exclusion. From 381 eligible residents, 273 randomly selected participants were approached for the sarcopenia study, with 102 (37.4%) providing written, informed consent. Additional exclusion criteria were then applied for the Activity Patterns Study: dementia (as determined by the service manager), non-ambulatory, and, unable to provide consent. All residents still eligible (n = 86; 84.3%) were invited to participate in the Activity Patterns Study. Ethical approval was obtained from the Human Ethics Committees of Uniting Care Queensland, Bond University and The University of Queensland.

### 2.2. Data Collection

The broader sarcopenia study used a single, one-hour assessment protocol including collection of socio-demographic and health measures (detailed below). For those in the Activity Patterns sub-study, the activity monitor was attached at the end of this assessment, with detailed instructions on how to replace the monitor if removed, and how to complete the sleep/wear diary. Qualitative, semi-structured telephone and in-person interviews were conducted with a key contact at each centre once monitors were returned. 

### 2.3. Measures

#### 2.3.1. Sociodemographic Measures

Age was calculated from date of birth, obtained from the aged care database. Data on gender, current smoking (yes–%), current alcohol consumption (yes–%), and education level (Secondary or higher–%) were obtained during the assessment.

#### 2.3.2. Physical Measures

Body Mass Index (BMI; kg∙m^−2^) was obtained by recording height and weight via a stadiometer and scales respectively. Information on falls in the previous 6 months (yes/no) was obtained both from self-report and from RAC facility records. The valid and reliable [16] Short Physical Performance Battery was used to measure lower body strength, balance, and walking ability [17]. Here, a higher score indicates higher lower body functioning (range 0–12). The aggregate score from this test was used as a measure of mobility.

#### 2.3.3. Cognitive Measures

The Mini Mental State Exam was used to measure cognition [18]. This test is useful in estimating an individual’s cognitive ability and incorporates questions ranging from registration of words to memory recall. The test has good reliability (test-retest and internal consistency) and construct validity [19]. Scores were coded ranging from moderate/severe cognitive impairment (0–18), mild impairment (19–24) and no impairment (25–30).

The Geriatric Depression Scale [20], which measures depression in the elderly, is a 30-item measure with yes/no questions. This scale has been found to be valid and reliable in both the general older adult population as well those in aged care [21]. Scores were coded ranging from severe depression (0–9), mild depression (10–19) and no depression (20–30).

#### 2.3.4. Activity Outcomes

Activity outcomes were measured by the valid and reliable [22] activPAL3^TM^ (version 6.3.1, default settings) activity monitor. This small (53 × 35 × 7 mm; 15 g), unobtrusive monitor classifies the raw activity data into periods spent sitting/lying, standing, and stepping (*i.e.*, walking), as well as recording step count. The activity monitor was waterproofed (Opsite Flexifix, Smith & Nephew Inc., Memphis, TN, USA), secured onto the right anterior mid-line of the right thigh with a hypoallergenic patch (Hypafix adhesive, Smith & Nephew Inc., Memphis, TN, USA), and worn 24 h/day for seven days. Participants recorded in a sleep/wear diary (see supplementary file) their awake/sleep times, monitor removal (if any), naps (if any), and any additional comments on their experience wearing the monitor. Nursing staff support was available for monitor assistance and diary completion. At the completion of the seven-day wear protocol, a nominated RAC facility staff member collected the monitors and posted them to the research team. 

#### 2.3.5. Evaluation of Feasibility

Feasibility of use of the activPAL3^TM^ monitors in the RAC setting was evaluated through quantitative (including consent rate; diary completion; monitor wear data) and qualitative (reasons for non-consent; open ended questions in the diary; semi-structured interviews) measures. Telephone and in-person interviews asked questions relating to both residents’ experiences (e.g., Did the residents have any complaints about the monitors?) and staff experiences (e.g., Did this process significantly impact on your ability to complete your daily tasks?). These included questions about the clarity of information provided regarding the study protocols, concerns with the monitors, and suggestions for future studies.

### 2.4. Data Processing

Monitor data were processed using SAS 9.3 (SAS Institute Inc., Cary, NC, USA) using customized programs that combined activPAL3^TM^ and diary data. For the 15-second epoch files, all times during self-reported sleeping or removal period were excluded while for the events (bouts) data, all bouts that were mostly (≥50%) asleep and/or removed were excluded. If not reported, apparent sleep/wake times were estimated based on visual scanning of the data for cessation/resumption of standing or stepping preceding/following prolonged periods of sitting or lying. Days were first defined based on night/day sleep-wake cycles then considered valid if wear time comprised ≥80% of waking hours. If waking hours were not reported, ≥10 h of wear time was considered a valid day. Sitting time accumulation data were derived by identifying all sitting bouts during waking, worn time on valid days, and calculating the cumulative sitting time (in minutes and as a proportion of total sitting time) occurring in bouts of up to each duration. To determine whether activity patterns varied across the day, activity outcomes were also reported by each hour using epoch files with outcomes reported as percentages of worn waking time. Hours were considered valid if ≥80% of the waking portion of these hours was wear time. Hours before 6 AM and after 9 PM were collapsed (<6 AM and >9 PM). 

### 2.5. Sample Size

With standard deviations of 80 min sitting, 60 min standing, and 25 min stepping, (assumed based on unpublished data from earlier activPAL^TM^ studies), it was determined that a sample size of 30 was required to place the confidence intervals (CI’s) around mean values to within±30 min for sitting and standing, and ±10 min for stepping. These variations in sitting, standing and stepping were considered meaningful for this population. Allowing for 30% missing data (e.g., due to non-compliance, monitor failure, *etc.*) approximately 40 participants would need to be recruited.

### 2.6. Statistical Analyses

Analyses were conducted using SPSS statistical software, version 21.0. Significance was set at *p* < 0.05 (two-tailed). Due to the low number of participants per centre (1 to 6), and the explorative nature of the study, analyses did not account for clustering. Descriptive statistics are presented as means and standard deviations for normally distributed data, median (interquartile range) for non-normal continuous data, or percentages for categories. Participant characteristics were described and compared with the RAC population in Australia (age and gender) [23] and eligible non-participants, using chi-square tests (categories), t-tests (normal data) or test of medians (non-normal data). Differences of ≥20% are reported. Residents’ activity patterns were examined in terms of average levels of activity outcomes (sitting, standing and stepping, corrected for wear by the residuals method), as well as step counts, using descriptive statistics. Sitting bout duration was examined using an accumulation curve. The accumulation curve depicts the observed proportion of total sitting time cumulatively accrued (Y-axis) in bouts of increasing duration (X-axis). The observed bout durations at which 10%, 50% and 90% of total sitting time were accrued were marked, as were the proportions of sedentary time accrued with commonly used cut-offs for prolonged sitting time (30 and 60 min) [24].

Linear mixed models were used to examine whether there were variations in activity by day of the week (time per day spent sitting, standing and stepping) or hour of the day (standing and stepping as percentages of wear time per hour). These analyses further adjusted for potential confounders (*i.e.*, variables of clinical interest that had an association with sitting, standing or stepping at *p* < 0.2; daily models also adjusted for wear time). Potential confounders tested included age, gender, cognitive impairment, depression, mobility, BMI, and falls. All outcomes were examined using a generalised linear mixed model (with normal distribution and identity link or gamma distribution and log link), which accounted for repeated measures. Models assumed distributions that best approximated the data: normal (sitting per day) or gamma (standing and stepping per day and per hour). None of the distributions were appropriate for sitting per hour. Models used whichever variance-covariance structure best fit the data (always compound symmetry).

The Spearman Brown Prediction Formula [24] was used to investigate how many days of monitoring are required to achieve a desired reliability (interclass correlation: ICC) of 0.8 and 0.9 for each activity outcome. ICCs were calculated from repeated measures ANOVAs of those with 7-days of data.

## 3. Results

### 3.1. Sample Characteristics

Table 1 illustrates the characteristics of the study participants and eligible non-participants. The sample ranged from 61.4 to 95.8 years of age. Consistent with the gender and age distribution in the broader Australian RAC population, the majority were women (65% *vs*. 72%, *p* = 0.65) and a third to half were aged 85 years and over (35% *vs*. 53%, *p* = 0.48) [23]. The sample predominantly consisted of those who did not currently smoke cigarettes or consume alcohol, were overweight or obese, had some form of cognitive impairment and depression, and very low mobility scores. Eligible non-participants were similar to participants and there were no significant predictors of participation. 

**Table 1:** Sociodemographic, Physical and Cognitive Characteristics of Activity Patterns Study (n = 31) Participants and Non-Participants (n = 45).

Characteristics		Activity Patterns Study (n = 31)	Eligible Non-Participants (n = 45)	*p* value
Age (years) mean		84.2	83.6	0.767
Women		20 (64.5%)	26 (57.8%)	0.273
Current smoker		7 (22.2%)	6 (13.3%)	0.458
Currently consume alcohol		6 (19.4%)	16 (35.6%)	0.203
Education–Secondary or higher		16 (51.6%)	27 (60.0%)	0.624
Body Mass Index (kg∙m^−2^) ^a^ mean (SD)		27.2 (5.7)	27.9 (5.6)	0.939
	Underweight (<18.5)	1 (3.0%)	0 (0.0%)	
	Normal (18.5–24.99)	10 (32.3%)	12 (27.9%)	
	Overweight (25.0–29.99)	11 (35.5%)	16 (37.2%)	
	Obese (≥30.0)	9 (29.0%)	15 (34.9%)	
Any falls in previous 6 months—Yes		11 (35.5%)	17 (37.8%)	0.837
Seniors Physical Performance Battery (0–12) ^b^		4.0 (1–9)	2.0 (0–11)	0.063
	Repeated Chair Stands (s) ^c^	21 (16.4–29.5)	22.5 (12.9–29.4)	
	Standing Balance (s)	20 (10–30)	20 (10–30)	
	Walking Speed (m∙s^−1^)	0.43 (0.14–0.75)	0.34 (0.02–1.0)	
Mini Mental State Exam (0–30)^ b^				0.468
	Moderate/Severe Cog Impairment (0–18)	9 (29.0%)	11 (24.4%)	
	Mild Cog Impairment (19–24)	8 (25.8%)	19 (42.2%)	
	No Cog Impairment (25–30)	14 (45.2%)	15 (33.3%)	
Geriatric Depression Scale (0–30)^ b^				0.552
	Severe (0–9)	19 (61.3%)	25 (55.6%)	
	Mild (10–19)	5 (16.1%)	8 (17.8%)	
	None (20–30)	7 (22.6%)	12 (26.7%)	

^a ^WHO BMI categories 2013; ^b ^higher scores indicate better performance; ^c ^n = 9 for participants; n = 10 for non-participants.

#### 3.1.1. Aim 1: Feasibility of Measurement

Of the 86 eligible from the broader sarcopenia study, 41 agreed to take part in this sub-study (47.8%). Most common reason for not consenting was “didn’t want to participate” (30 of 45 cases). Other reasons included “believe it will be too difficult” (8 of 45 cases), “my doctor doesn’t want me to participate (6 of 45 cases), and “I don’t see a benefit” (1 of 45 cases). During the course of the study, one participant was found to be ineligible (mobility impairment), three participants provided no valid data (less than 1 day), and six monitors were lost (no monitors failed). Thirty-one participants were included in the final analyses (75.6%), of which the majority (n = 26; 83.9%) provided seven days of data, while two, one, and two provided six, three, and one days of data, respectively. 

With respect to diary completion, two were not returned, two were blank, 13 were partially completed, and 14 were completed in full. Five provided at least one comment regarding wearing the monitor with consistently positive feedback. Informal telephone interviews were achieved with nine (of 11) RAC centres. 

Through the various interviews conducted (described above) it was determined that residents were not burdened by wearing the monitor. Importantly, no residents experienced skin irritation due to the monitor adhesive. Telephone interviews with staff suggested that the study protocols did not adversely affect their work schedule. However, staff members commented they did not have the time to give the required attention to the study participants to answer all questions (help filling out diary, general discussion). Staff comments for improvement in future studies included incorporating staff members in the study design process, encouraging the circulation of study protocols to all staff members, and suggestions for simplifying the sleep diary. Notably, these included devising a one-page version, as opposed to booklet-form, and only including sections on sleep/wake time (including naps). 

#### 3.1.2. Aim 2: Activity Patterns

##### Overall Activity Patterns

The average (mean ± SD) worn waking hours were 14.6 ± 2.0 h. Average (median, 95% CI) levels of activity in aged care residents show that, standardised for worn waking time, most waking hours are spent sitting/lying (12.4, 95% CI = 11.3 to 13.3 h; interquartile range = 1.7 h) followed by standing (1.9, 95% CI = 1.2 to 2.6 h; interquartile range = 1.3 h). Very little time is spent stepping (21.4, 95% CI = 11.2 to 36.7 min; interquartile range = 23.8 min), while mean daily step count is generally low (1055, 95% CI = 335 to 1,768 steps, interquartile range = 1,110 steps).

**Figure 1:**
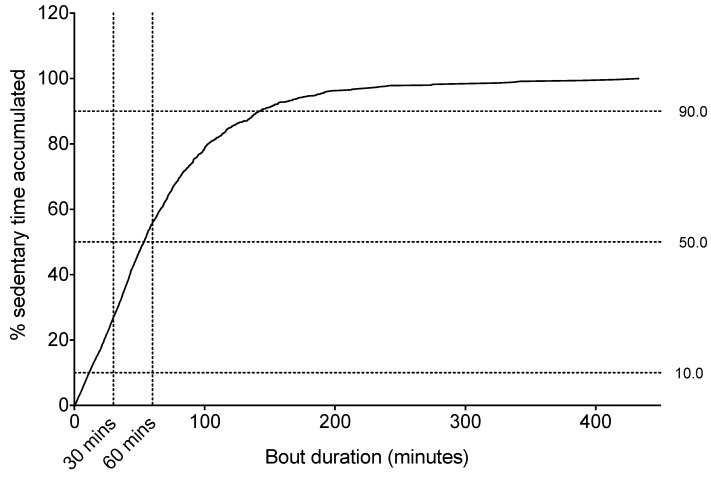
A sedentary accumulation graph depicting the cumulative percentage of sitting time in relation to bout duration. Lines indicating the 10th, 50th, and 90th percentiles are highlighted. Cumulative percentage of sitting time is also depicted for the common cut-offs of 30 and 60 min.

##### Sitting Time Accrual

Figure 1 shows the accumulation of sitting time in relation to the duration of sitting bouts. Excluding the top and bottom 10%, the majority (80%) of sitting time is accrued across a wide range of bout durations that includes very long bouts: approximately 11 to 142 min. Half of all sedentary time was accrued in bouts of ≥53 min; the other half was accumulated in bouts shorter than this duration. Respectively, approximately 73% and 44% of sitting time was accumulated in bouts ≥30 min and ≥60 min in duration. 

**Figure 2:**
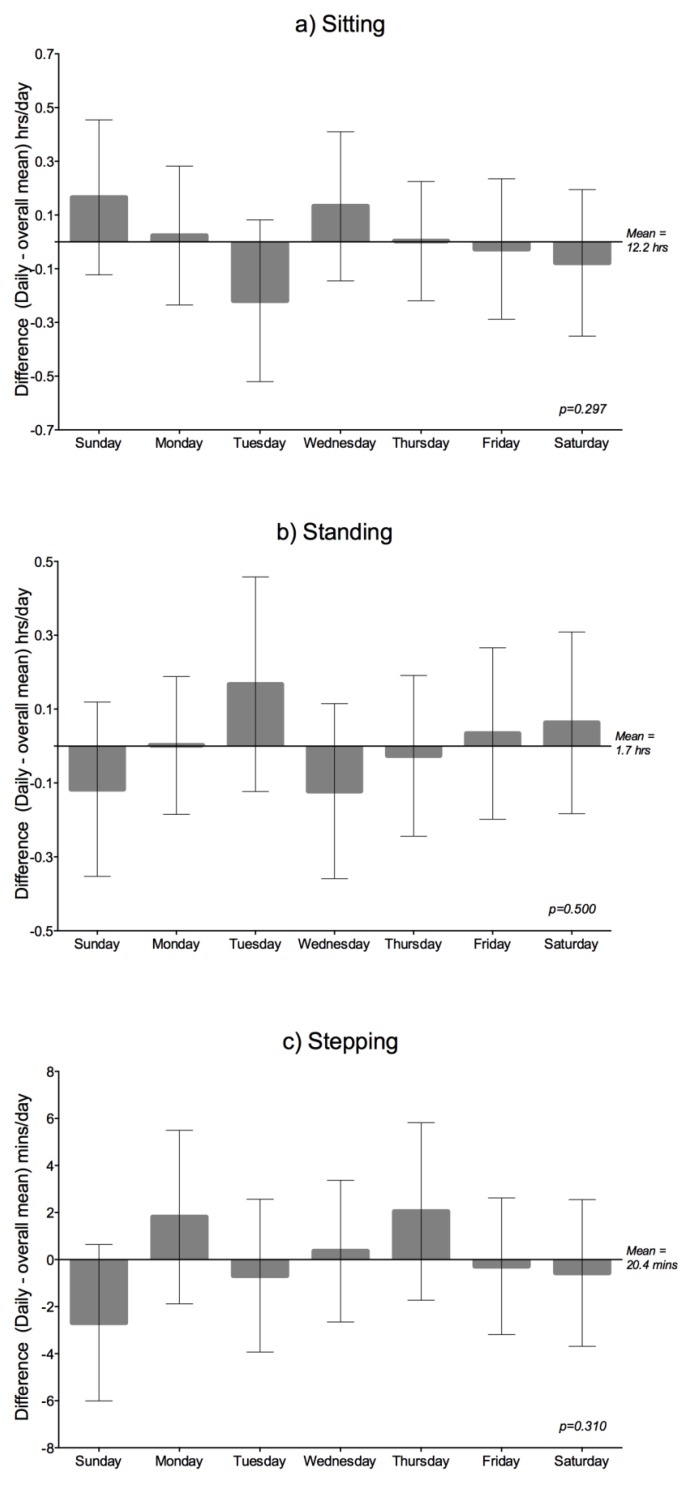
Average (mean, 95% CI) time spent (**a**) Sitting (**b**) Standing and (**c**) Stepping for 31 adults in residential aged care. Twenty-eight observations were available for Sunday, Monday, Tuesday, and Friday, while 29 were available for the remaining days. The sitting time and stepping time analyses included age, gender, cognitive impairment, depression, mobility and falls as covariates, whereas the walking time analysis included only cognitive impairment.

##### Daily Variation

Figure 2 shows how the adjusted mean time spent sitting, standing, and stepping across each day of the week departs from the overall adjusted mean. Here, no statistically significant differences between days of the week were observed for any of the activity outcomes. Further, the 95% CI’s excluded as unlikely any meaningful differences (*i.e.*, ≥±30 min for sitting/standing and ≥±10 min for stepping). Day to day variability (ICCs) and the number of days of monitoring required to reliably measure activity outcomes were investigated. To achieve ICCs of 0.8 to 0.9, five to 11 days are needed for sitting, five to ten days are required for standing, while seven to 15 days are necessary for stepping. 

**Figure 3:**
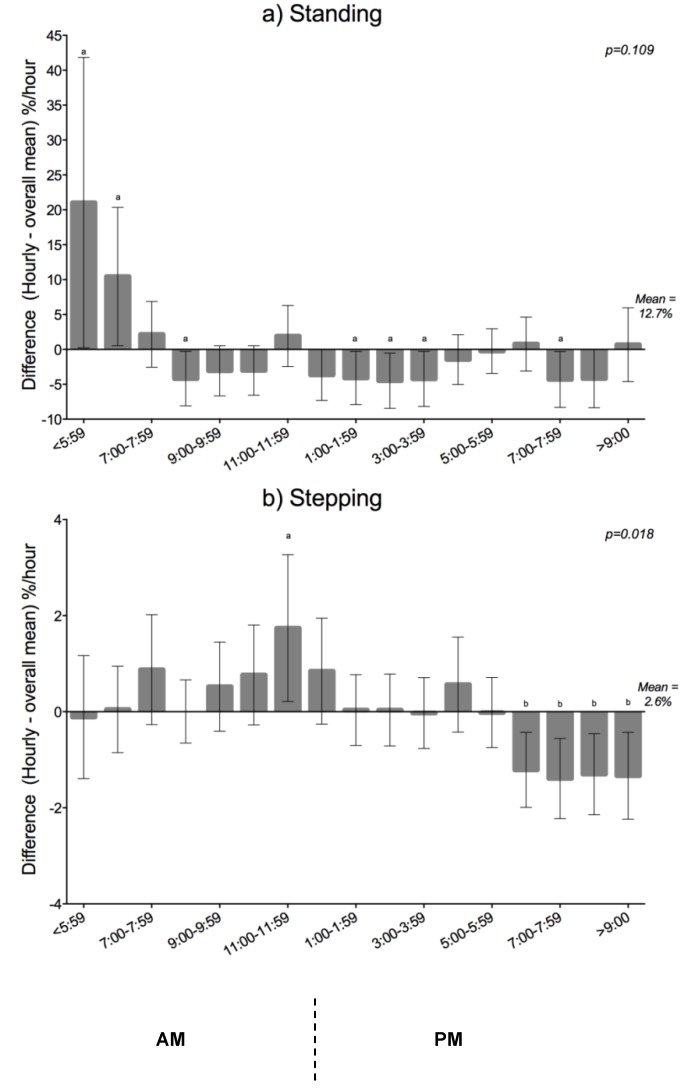
Average (mean, 95% CI) percent of time each hour spent (**a**) Standing, and (**b**) Stepping compared to the overall mean percentage in 31 adults in residential aged care. Depression and falls were entered as significant covariates in both models. The standing time analysis further included age, gender, cognitive impairment, and mobility as covariates. Note: 9 = 09:00 to 09:59 AM, <5:59 AM all collapsed and 9 PM+ all collapsed. ^a^ denotes *p* < 0.05; ^b^ denotes *p* < 0.001.

##### Hourly Variation

Figure 3 shows the adjusted mean percentage of time spent standing and stepping across each hour of the day compared with the overall adjusted percentage. The overall model for standing per hour was not significant; however, individual hours varied significantly from the overall adjusted mean: more standing occurred up to 7 AM, less between 8 to 9 AM, 1 to 4 PM, and 7 to 8 PM. Stepping varied significantly, with more stepping occurring between 11 AM and 12 PM, and less after 6 PM.

## 4. Discussion

This study described the feasibility of investigating the activity patterns (sitting, standing and stepping) of older adults in residential aged care using device-based measurement and examined overall time in these activities, variation across days and hours, and how sitting time was accrued. Device-based activity monitors (7-day continuous wear protocol) are feasible to use in this population, albeit with several considerations. Monitor data indicated that older adults in RAC are highly sedentary, spending an average 85% of waking hours sitting or lying, with nearly half of this sedentary time accrued in prolonged, unbroken bouts of at least 60 min. Moreover, similar to what has been observed in hospital ward patients, there was minimal variation in sedentary time across or within days. 

There were no adverse consequences of activity monitoring. The consent rate was relatively high (47.8%) [25]; however, as participants were recruited from a larger study, this rate should be interpreted accordingly. Of the 31 (of 41 cases) participants with any valid data, 26 provided seven days of data, with 14 fully completing the diary, while six monitors were lost. Suggested improvements to enhance compliance include increasing RAC staff involvement (including increased training and education) and simplifying the diary to a single page. Findings did not contradict previous literature [26], showing the minimum requirement to obtain reliable estimates in older adults to be five days required for estimating sitting and standing time, and seven days of monitoring for stepping. 

The average sitting time in this sample (>12 h/day; 85%) exceeds that observed in previous studies in healthy, community-dwelling older adults which have reported accelerometer-assessed average sedentary times ranging from 8.4 to 11 h [3,8,27] per day. No studies have previously compared the activity patterns of RAC and community-dwelling older adults. Although differences in sitting time could be due to use of different measurement devices, findings from previous studies indicate that differences are unlikely due to factors such as age. Another possible explanation is that those that enter residential aged care are often suffering from debilitating health problems that impair their movement. Such conditions, which are precursors to institutionalization and highly prevalent in residential aged care, include sarcopenia [28] and stroke [29]. Further studies using standardized measures are required to determine the true prevalence of sedentary behavior in the residential aged care population. Daily standing and stepping time were lower than previously observed. Although not directly comparable to activPAL data, accelerometer findings show community-dwelling older adults spend approximately 21% of daily waking time in light-intensity activity (including standing) [8], and less than 2% in MVPA [30]. Lastly, average daily step counts (1,055 per day) were very low. Although some misclassification of step number at very slow speeds is possible, this would still have been detected as upright time and not influenced overall sitting time. 

A unique element of this study is that it not only looked at total sedentary time, but also how the sedentary time was accumulated. Here, over 70% of total sitting time was accrued in bouts of 30 min or more, with nearly 50% accrued in bouts of at least 60 min. This is comparable to a study using the same monitor in a sample of hospital ward physiotherapy patients [31]. Such prolonged, unbroken sitting time increases the risk for musculoskeletal problems [32,33,34,35], loss of physical function [36] and mobility. Conversely, recent studies have shown acute cardiometabolic benefits of regularly interrupting sitting time [37,38], with physical function and/or falls rates also significantly improved in older adults by embedding regular physical activities such as the sit-stand into their daily routine [25,27]. Research is needed to evaluate the bout durations used to define prolonged sitting for this population, as well as the feasibility, and health benefits, of regularly interrupting it whilst addressing staff needs, falls risk and compliance. 

Statistically significant differences in hourly standing and stepping were found. Generally, this population was more active in the mornings, with lowered activity from 1 PM onwards. Accordingly, interventions in RAC populations have scope to address behaviour across the day, but should consider fatigue-related compensation by targeting times unlikely to interfere with current periods of greatest standing and stepping (up to 12 PM).

Strengths of this study include detailed objective, 24-h measurement of activity using a valid and reliable monitor. The use of an events-based approach and the integration of corroborating self-report information are also key methodological advances from previous research relying on the less precise epoch files and/or unverified assumptions regarding sleep and non-wear [39]. Finally, analyses corrected for potentially confounding variables. A primary limitation of this study was the small sample size. The study was not powered *a priori* for detecting daily or hourly variation, however mostly, the sample size was adequate to yield conclusive results—the 95% CI’s did not encompass meaningful effects. The sample size was insufficient to stratify by potential key descriptors (e.g., including depression, age, disease and mobility) or to correct for or examine the influence of the RAC centre (including the availability of physical activity programs). Therefore, it was not possible to determine what influence these descriptors had on sedentary time. Further limitations are observed with possible sampling issues, misclassification of sleep/wake time (particularly for naps of short duration), and in the validity of the activPAL3^TM^. Although those with dementia as identified by the service manager were excluded, nine participants were still identified as having severe cognitive impairment. However, cognitive status was adjusted for in each model. Lastly, although the uniaxial activPAL has been validated in older adults [22], it is important to note that the triaxial activPAL3^TM^ has not.

## 5. Conclusions

This was the first study to measure activity and sedentary patterns in older adults in RAC, as well as to evaluate the feasibility of using device-based measures in this population. The findings have highlighted that this population is more sedentary than community dwelling older adults and exhibits limited systematic variation between days and small variation within the day. While it is of little importance which days are monitored, the recommended monitoring period should be at least one or two weeks for this population, depending on the required measures and level of repeatability. Further investigation is required to inform interventions, including, but not limited to, determining appropriate definitions of prolonged sitting, and safe and acceptable levels of change that lead to health benefits for this population.

## Supplementary Files

Supplementary File 1 Supplementary (PDF, 180 KB)
