# Supplementary material for: Objectively Measured Activity Patterns among Adults in Residential Aged Care
Source: Int J Environ Res Public Health. 2013 Dec 4;10(12):6783–98. doi: 10.3390/ijerph10126783 (PMC3881141; doi:10.3390/ijerph10126783)
Supplement: Supplementary File 1 — Supplementary (PDF, 180 KB) [file ijerph-10-06783-s001.pdf]

COMMENTS

Please write any comments you think might be useful for this study. Particularly include any information on how easy or difficult you found it to wear the activity monitor and/or fill out this sleep diary. This information will be used to inform future studies.

If you have any questions, contact Natasha Begovic on (07) 3365 5163 or 0411 039 969

THANK YOU VERY MUCH FOR PARTICIPATING

THE UNIVERSITY OF QUEENSLAND AUSTRALIA

ID: C U S _ _ _ _ _ _ _

Monitor Pack Number: ☐☐☐

activPAL serial # __ __ __ __ __ __

SARCOPENIA AMONG OLDER AUSTRALIANS

Activity Monitor Instructions

& Sleep Diary

Please keep this booklet in a safe place so you can return it to us

Day 1

____/____/____

If you have any questions or concerns,

please contact your nurse or Natasha Begovic on

0411 039 969 or email: n.begovic@uq.edu.au

Nap 2	Notes
Start: _______ am/pm<br>Finish: _______ am/pm	
Start: _______ am/pm<br>Finish: _______ am/pm	
Start: _______ am/pm<br>Finish: _______ am/pm	
Start: _______ am/pm<br>Finish: _______ am/pm	
Start: _______ am/pm<br>Finish: _______ am/pm	
Start: _______ am/pm<br>Finish: _______ am/pm	
Start: _______ am/pm<br>Finish: _______ am/pm	
Start: _______ am/pm<br>Finish: _______ am/pm	

Day	Did you have any naps today?	Nap 1
Example<br>25th July, 2012	Yes ☐<br>No ☐	Start: 3:00 am/pm (pm circled)<br>Finish: 4:30 am/pm (pm circled)
Day 1	Yes ☐<br>No ☐	Start: ______ am/pm<br>Finish: ______ am/pm
Day 2	Yes ☐<br>No ☐	Start: ______ am/pm<br>Finish: ______ am/pm
Day 3	Yes ☐<br>No ☐	Start: ______ am/pm<br>Finish: ______ am/pm
Day 4	Yes ☐<br>No ☐	Start: ______ am/pm<br>Finish: ______ am/pm
Day 5	Yes ☐<br>No ☐	Start: ______ am/pm<br>Finish: ______ am/pm
Day 6	Yes ☐<br>No ☐	Start: ______ am/pm<br>Finish: ______ am/pm
Day 7	Yes ☐<br>No ☐	Start: ______ am/pm<br>Finish: ______ am/pm

INSTRUCTIONS

How to fill in this diary

Wake Time:

In this section, please write the time you actually woke up, not the time you got out of bed. For example, if you woke up at 7am, but did not get out of bed until 8am, please record your wake time as 7am. Please note if it was am or pm.

Sleep Time:

In this section please indicate what time you actually fell asleep. As before, please do not record the time you went to bed. For example, if you went to bed at 8pm, read for half an hour and fell asleep at 8:30pm, please record your sleep time as 8:30pm. Again, please note if it was am or pm.

**Date**	**Wake time**	**Sleep time**	**Did you go outside in daylight hours for more than 15 minutes today?**	**Did you remove the monitor for more than 10 minutes today?** Please cross box	**If yes, when did you remove it?**
Example 25th July, 2012	7:15 am/pm	9:25 am/pm	Yes ☐ No ☐	Yes ☐ No ☐	Off: _______ am/pm On: _______ am/pm
Day 1	am/pm	am/pm	Yes ☐ No ☐	Yes ☐ No ☐	Off: _______ am/pm On: _______ am/pm
Day 2	am/pm	am/pm	Yes ☐ No ☐	Yes ☐ No ☐	Off: _______ am/pm On: _______ am/pm
Day 3	am/pm	am/pm	Yes ☐ No ☐	Yes ☐ No ☐	Off: _______ am/pm On: _______ am/pm
Day 4	am/pm	am/pm	Yes ☐ No ☐	Yes ☐ No ☐	Off: _______ am/pm On: _______ am/pm
Day 5	am/pm	am/pm	Yes ☐ No ☐	Yes ☐ No ☐	Off: _______ am/pm On: _______ am/pm
Day 6	am/pm	am/pm	Yes ☐ No ☐	Yes ☐ No ☐	Off: _______ am/pm On: _______ am/pm
Day 7	am/pm	am/pm	Yes ☐ No ☐	Yes ☐ No ☐	Off: _______ am/pm On: _______ am/pm

If you took any naps please write them on the next page

If you have any comments please write them on the last page
